# Wounds in War

**Published:** 1899-04

**Authors:** 


WOUNDS IN WAR. The mechanism of their production and their treatment. By Surgeon-Colonel W. F. Stevenson (Army Medical Staff), A. B., M. B., M. Ch., Dublin University; Professor of Military Surgery, Army Medical School, Netley. Published by Wm. Wood & Co., New York, 1898. Price, Cloth, $4.00 net; 437 pages.

This work comes from such a source as would indicate it as an official deliverance of surgical standards for the British army. It is technical in the extreme, but full of good things. It might be as well entitled a treatise on gunshot wounds, however, as this is the main part of its contents.

In fact this is about all that is left of military surgery, since the improvements in firearms have practically abolished sword, lance and bayonet wounds. Almost all of these wounds recorded in recent wars, have been caused by camp fights, between soldiers of the same army.

Almost one-third of the work is devoted to a technical description of the various firearms and ammunition in use by the different nations, and the peculiarity of wounds inflicted by each. In this enumeration I fail to find our own country mentioned, but I suppose it was taken for granted that in the hands of the born soldiers and marksmen of this country any old gun would do.

The author makes a strong plea for the specialty of military surgery, which I cannot endorse. From the medical and surgical histories of wars that I have studied, it would seem to me that what is more needed is a specialist on sanitation and hygiene with power to override red tape.

Our younger physicians should read this and similar treatises at this time, if, as seems inevitable, our country is to embark in an extensive military policy. There will be a great demand for young surgeons in the medical department, and these are splendid positions.

I note with pleasure the following tribute to one product of our own army medical corps: "I have freely availed myself of that inexhaustible source of information upon this subject, 'The Surgical History of the War of the Rebellion' in the United States, by G. A. Otis, a work which must continue to be a record of labor and research which may never be surpassed." I. J. J.

